# Supplementary material for: Key Taxa of the Gut Microbiome Associated with the Relationship Between Environmental Sensitivity and Inflammation-Related Biomarkers
Source: Microorganisms. 2025 Jan 16;13(1):185. doi: 10.3390/microorganisms13010185 (PMC11767568; doi:10.3390/microorganisms13010185)
Supplement: Supplementary file 1 [file microorganisms-13-00185-s001.zip › microorganisms-3395404-supplementary.pdf]

Supplementary materials

The genus *Butyricimonas* is the only taxa for which a significant interaction effect was observed in relation to CRP. Additionally, for the family, *Marinifilaceae*, which includes the genus *Butyricimonas*, a trend toward an interaction effect (p = 0.062) was noted in association with LBP. Therefore, we conducted a hierarchical multiple regression analysis focused on LBP for this taxon. As indicated in supplementary Table S1, we observed similar results for the genus *Butyricimonas* (ΔR^2 = 0.031, p = 0.056), as well as for the genus *Coprobacter*, genus *Barnesiella*, genus *Akkermansia*, genus *Family XIII AD3011 group*, genus *GCA-900066225*, and genus *Ruminiclostridium 1*.

Supplementary Table S1. Interaction effects between environmental sensitivity and the genus *Butyricimonas* predicting LBP (n = 88).

	Log-normalized LBP			
	Step1		Step2	
Predictors	β	p	β	p
Age	0.034		0.043	
Sex	0.003		-0.012	
BMI	0.509	**	0.488	**
HSP-J10	0.128		0.162	†
Genus *Butyricimonas*	-0.039		-0.074	
HSP-J10 × Genus *Butyricimonas*			-0.185	†
R^2	0.290	**	0.322	**
ΔR^2			0.031	†

Note. ** $p < 0.01$; † $p < 0.10$

LBP: lipopolysaccharide-binding protein; BMI: body mass index; HSP-J10: environmental sensitivity.

Next, we conducted simple slope tests on the genus *Butyricimonas* in relation to LBP. Individuals with high environmental sensitivity exhibited no association with LBP when the abundance of *Butyricimonas* was high (M+1SD; β = -0.004, p = 0.969). In contrast, LBP levels were significantly elevated when the abundance of *Butyricimonas* was low (M-1SD; β = 0.320, p = 0.022) (Supplementary Figure S1).

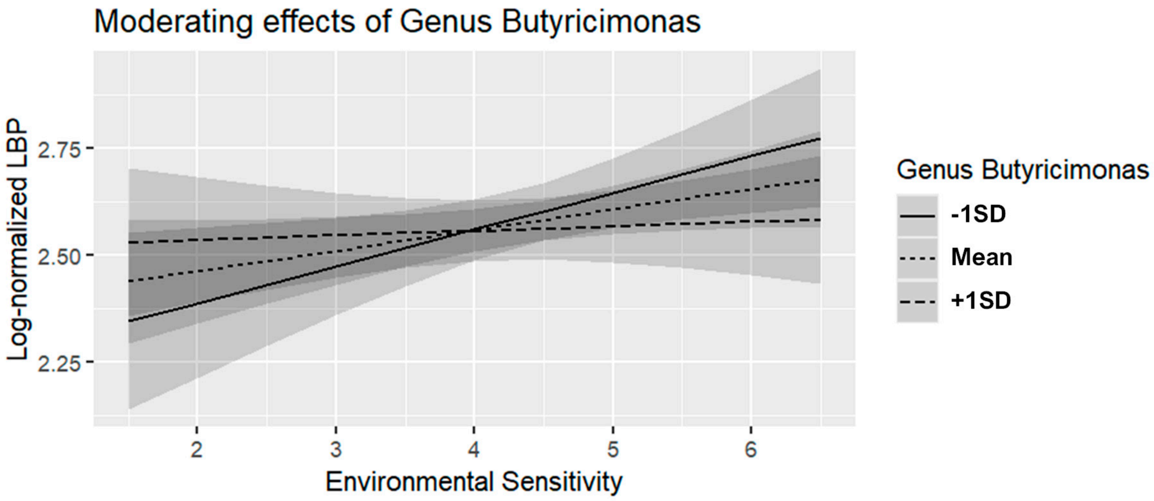

Supplementary Figure S1. Moderating effects of the genus *Butyricimonas* on the relationship between environmental sensitivity and lipopolysaccharide-binding protein (LBP) ($n = 88$).

As an exploratory analysis, we examined interaction effects between environmental sensitivity and specific taxa using hierarchical multiple regression analysis in a similar manner by adding frequencies of vegetable and fruit intakes as independent variables. Frequencies of vegetable and fruit intakes were assessed by using a food frequency questionnaire, respectively. As indicated in Supplementary Tables S2 and S3, for CRP, similar results were observed for the family *Marinifilaceae* ($\Delta R^2 = 0.026$, $p = 0.071$) and the genus *Butyricimonas* ($\Delta R^2 = 0.037$, $p < 0.05$), even when the frequencies of vegetable and fruit intakes were entered as independent variables. For LBP, similar results were observed for the family *Barnesiellaceae* ($\Delta R^2 = 0.030$, $p = 0.057$) (Supplementary Table S4), the family *Akkermansiaceae* ($\Delta R^2 = 0.029$, $p = 0.063$) (Supplementary Table S5), the genus *Coprobacter* ($\Delta R^2 = 0.030$, $p = 0.056$) (Supplementary Table S6), the genus *Akkermansia* ($\Delta R^2 = 0.029$, $p = 0.063$) (Supplementary Table S7), the genus *Family XIII AD3011 group* ($\Delta R^2 = 0.050$, $p < 0.05$) (Supplementary Table S8), the genus *GCA-900066225* ($\Delta R^2 = 0.044$, $p < 0.05$) (Supplementary Table S9) , and the genus *Ruminiclostridium 1* ($\Delta R^2 = 0.028$, $p = 0.066$) (Supplementary Table S10).

Supplementary Table S2. Interaction effects between environmental sensitivity and family *Marinifilaceae* predicting CRP ($n = 88$).

	Log-normalized CRP			
	Step1		Step2	
Predictors	*β*	*p*	*β*	*p*
Age	-0.148		-0.161	†
Sex	-0.125		-0.140	
BMI	0.557	**	0.550	**

Vegetable intake	0.130		0.116	
Fruit intake	-0.113		-0.080	
HSP-J10	0.196	*	0.187	*
Family *Marinifilaceae*	0.011		0.026	
HSP-J10 × Family *Marinifilaceae*			-0.166	†
R^2	0.361	**	0.387	**
ΔR^2			0.026	†

Note. ** $p < 0.01$; * $p < 0.05$; † $p < 0.10$. CRP: C-reactive protein; BMI: body mass index; HSP-J10: environmental sensitivity.

Supplementary Table S3. Interaction effects between environmental sensitivity and genus *Butyricimonas* predicting CRP ($n = 88$).

	Log-normalized CRP			
	Step1		Step2	
Predictors	*β*	*p*	*β*	*p*
Age	-0.147		-0.137	
Sex	-0.129		-0.138	
BMI	0.556	**	0.530	**
Vegetable intake	0.132		0.107	
Fruit intake	-0.114		-0.099	
HSP-J10	0.197	*	0.230	*
Genus *Butyricimonas*	0.018		-0.022	
HSP-J10 × Genus *Butyricimonas*			-0.204	*
R^2	0.361	**	0.399	**
ΔR^2			0.037	*

Note. ** $p < 0.01$; * $p < 0.05$. CRP: C-reactive protein; BMI: body mass index; HSP-J10: environmental sensitivity.

Supplementary Table S4. Interaction effects between environmental sensitivity and family *Barnesiellaceae* predicting LBP ($n = 88$).

	Log-normalized LBP			
	Step1		Step2	
Predictors	*β*	*p*	*β*	*p*
Age	0.067		0.082	
Sex	-0.035		-0.038	
BMI	0.515	**	0.455	**
Vegetable intake	0.038		0.027	

Fruit intake	-0.204	*	-0.186	†
HSP-J10	0.166	†	0.146	
Family *Barnesiellaceae*	0.112		0.123	
HSP-J10 × Family *Barnesiellaceae*			-0.183	†
R^2	0.342	**	0.371	**
ΔR^2			0.030	†

Note. ** $p < 0.01$; * $p < 0.05$; † $p < 0.10$. LBP: lipopolysaccharide-binding protein; BMI: body mass index; HSP-J10: environmental sensitivity.

Supplementary Table S5. Interaction effects between environmental sensitivity and family *Akkermansiaceae* predicting LBP ($n = 88$).

	Log-normalized LBP			
	Step1		Step2	
Predictors	*β*	*p*	*β*	*p*
Age	0.071		0.061	
Sex	-0.037		-0.042	
BMI	0.515	**	0.532	**
Vegetable intake	0.064		0.050	
Fruit intake	-0.218	*	-0.202	*
HSP-J10	0.166	†	0.141	
Family *Akkermansiaceae*	0.044		0.070	
HSP-J10 × Family *Akkermansiaceae*			-0.175	†
R^2	0.332	**	0.360	**
ΔR^2			0.029	†

Note. ** $p < 0.01$; * $p < 0.05$; † $p < 0.10$. LBP: lipopolysaccharide-binding protein; BMI: body mass index; HSP-J10: environmental sensitivity.

Supplementary Table S6. Interaction effects between environmental sensitivity and genus *Coprobacter* predicting LBP ($n = 88$).

	Log-normalized LBP			
	Step1		Step2	
Variables	*β*	*p*	*β*	*p*
Age	0.065		0.018	
Sex	-0.021		-0.030	
BMI	0.520	**	0.500	**
Vegetable intake	0.062		0.078	
Fruit intake	-0.214	*	-0.183	†

HSP-J10	0.165	†	0.188	†
Genus *Coprobacter*	0.006		-0.079	
HSP-J10 × Genus *Coprobacter*			-0.203	†
R^2	0.330	**	0.361	**
ΔR^2			0.030	†

Note. ** $p < 0.01$; * $p < 0.05$; † $p < 0.10$. LBP: lipopolysaccharide-binding protein; BMI: body mass index; HSP-J10: environmental sensitivity.

Supplementary Table S7. Interaction effects between environmental sensitivity and genus *Akkermansia* predicting LBP ($n = 88$).

	Log-normalized LBP			
	Step1		Step2	
Predictors	*β*	*p*	*β*	*p*
Age	0.071		0.061	
Sex	-0.037		-0.042	
BMI	0.515	**	0.532	**
Vegetable intake	0.064		0.050	
Fruit intake	-0.218	*	-0.202	*
HSP-J10	0.166	†	0.141	
Genus *Akkermansia*	0.044		0.070	
HSP-J10 × Genus *Akkermansia*			-0.175	†
R^2	0.332	**	0.360	**
ΔR^2			0.029	†

Note. ** $p < 0.01$; * $p < 0.05$; † $p < 0.10$. LBP: lipopolysaccharide-binding protein; BMI: body mass index; HSP-J10: environmental sensitivity.

Supplementary Table S8. Interaction effects between environmental sensitivity and genus *Family XIII AD3011 group* predicting LBP ($n = 88$).

	Log-normalized LBP			
	Step1		Step2	
Predictors	*β*	*p*	*β*	*p*
Age	0.071		0.012	
Sex	0.009		0.010	
BMI	0.503	**	0.566	**
Vegetable intake	0.045		0.056	
Fruit intake	-0.210	*	-0.202	*
HSP-J10	0.175	†	0.162	†

Genus *Family XIII AD3011 group*	-0.094		0.071	
HSP-J10 × Genus *Family XIII AD3011 group*			-0.276	*
R^2	0.337	**	0.387	**
ΔR^2			0.050	*

Note. ** $p < 0.01$; * $p < 0.05$; † $p < 0.10$. LBP: lipopolysaccharide-binding protein; BMI: body mass index; HSP-J10: environmental sensitivity.

Supplementary Table S9. Interaction effects between environmental sensitivity and genus *GCA-900066225* predicting LBP ($n = 88$).

	Log-normalized LBP			
	Step1		Step2	
Predictors	*β*	*p*	*β*	*p*
Age	0.057		0.027	
Sex	-0.006		-0.033	
BMI	0.511	**	0.532	**
Vegetable intake	0.063		0.056	
Fruit intake	-0.215	*	-0.219	*
HSP-J10	0.180	†	0.122	
Genus *GCA-900066225*	-0.121		-0.049	
HSP-J10 × Genus *GCA-900066225*			-0.233	*
R^2	0.344	**	0.388	**
ΔR^2			0.044	*

Note. ** $p < 0.01$; * $p < 0.05$; † $p < 0.10$. LBP: lipopolysaccharide-binding protein; BMI: body mass index; HSP-J10: environmental sensitivity.

Supplementary Table S10. Interaction effects between environmental sensitivity and genus *Ruminiclostridium 1* predicting LBP ($n = 88$).

	Log-normalized LBP			
	Step1		Step2	
Predictors	*β*	*p*	*β*	*p*
Age	0.064		0.069	
Sex	-0.015		-0.038	
BMI	0.520	**	0.517	**
Vegetable intake	0.066		0.062	
Fruit intake	-0.221	*	-0.204	*
HSP-J10	0.163	†	0.145	
Genus *Ruminiclostridium 1*	-0.028		-0.101	

HSP-J10 × Genus *Ruminiclostridium 1*			-0.189	†
R^2	0.331	**	0.359	**
ΔR^2			0.028	†

Note. ** $p < 0.01$; * $p < 0.05$; † $p < 0.10$. LBP: lipopolysaccharide-binding protein; BMI: body mass index; HSP-J10: environmental sensitivity.
